# Assessment of short-term outcome with TiO_2_ mesh in laparoscopic repair of large paraesophageal hiatal hernias

**DOI:** 10.1186/s12893-019-0607-4

**Published:** 2019-10-28

**Authors:** Islam Khaled, Pablo Priego, Mohammed Faisal, Marta Cuadrado, Francisca García-Moreno, Araceli Ballestero, Julio Galindo, Eduardo Lobo

**Affiliations:** 1 Department of Surgery, Suez Canal University Hospitals and Medical School, Ismailia, Egypt; 2 Division of Esophagogastric, Bariatric and Minimally Invasive Surgery, Department of Surgery, Ramon y Cajal University Hospital, Crta. Colmenar Viejo Km 9,100, 28034 Madrid, Spain

**Keywords:** Hiatal hernia, Para-oesophageal hernia, Laparoscopic anti-reflux surgery, Synthetic mesh, TiO_2_Mesh™

## Abstract

**Background:**

Laparoscopic large para-oesophageal hiatal hernia (LPHH) repair using mesh reinforcement significantly reduces postoperative recurrence rates compared to conventional suture repair, especially within short follow-up times. However, the ideal strategy for repairing LPHH remains disputable because no clear guidelines are given regarding indications, mesh type, shape or position. The aim of this study was to survey our short-term results of LPHH management with a biosynthetic monofilament polypropylene mesh coated with titanium dioxide to enhance biocompatibility (TiO_2_Mesh™).

**Methods:**

A retrospective study was performed at Ramon y Cajal University Hospital, Spain from December 2014 to October 2018. Data were collected on 27 consecutive patients with extensive hiatal hernia defects greater than 5 cm for which a laparoscopic repair was performed by primary suture and additional reinforcement with a TiO_2_Mesh™. Study outcomes were investigated, including clinical and radiological recurrences, dysphagia and mesh-related drawbacks.

**Results:**

Twenty-seven patients were included in our analysis; 10 patients were male, and 17 were female. The mean age was 73 years (range, 63–79 years). All operations were performed laparoscopically. The median postoperative hospital stay was 3 days. After a mean follow-up of 18 months (range, 8-29 months), only 3 patients developed clinical recurrence of reflux symptoms (11%), and 2 had radiological recurrences (7%). No mesh-related complications occurred.

**Conclusions:**

TiO_2_Mesh™ was found to be safe for laparoscopic repair of LPHH with a fairly low recurrence rate in this short-term study. Long-term studies conducted over a period of years with large sample sizes will be essential for confirming whether this mesh is suitable as a standard method of care with few drawbacks.

## Background

Large para-oesophageal hiatal hernia (LPHH) repair remains a controversial and challenging intervention for most practising surgeons [1, 2]. Clinical and/or radiological recurrence after conventional repair using sutures occurs in up to 33% of patients, even with good clinical outcome [3].

The standard method of repairing LPHH is still debatable, because although short-term follow-up revealed that mesh-reinforced repair critically reduces the recurrence rate compared to conventional repair with sutures [4–8], no clear guidelines are given regarding indications, mesh type, shape and position [9, 10].

The drawbacks of using mesh in LPHH repair include a prolonged procedure time for the application and safe fixation of the mesh as well as complications related directly to the mesh, such as oesophageal erosion and postoperative dysphagia [11–13]. A group of surgeons have considered avoiding the use of synthetic mesh [e.g., polytetrafluoroethylene (PTFE), polypropylene, …] and shifting to new type of biologic and synthetic bio-absorbable meshes (e.g., intestinal submucosa, cadaveric human skin, bovine pericardium, collagen cross-linked mesh) to prevent or minimize complications [14–22]. However, the short- and long-term results of these studies were not as satisfactory as previously imagined [23].

Currently, an ideal mesh for hiatal repair does not exist. Despite the availability of different brands, almost all brands use one of the same three basic components: polyester, polypropylene, and PTFE. Differences between brands were based on their combination with each other or the addition of extra substances such as omega 3 fatty acids, titanium, hyaluronate, and poliglecaprone 25. Numerous standards exist, and measures of a proper synthetic mesh should be assured, such as having optimal biocompatibility and being malleable with adequate strength in order to avoid recurrence, degeneration or retraction. To fulfil these standards, ultra-lightweight, large-pore meshes have been developed recently, while considering the need for stability [24]. Previous experimental studies have shown that addition of an extra layer of atomic titanium to the polypropylene filaments (TiO_2_Mesh™) has led to further enhancement of the biocompatibility and a significant reduction in shrinkage rates in comparison to the same mesh without a covering of titanium [25].

To date, no clinical randomized studies have been conducted on TiO2Mesh™ for the repair of hiatal hernias, and therefore, no scientific declarations can be provided about this type of mesh. The other available analyses in the literature relate exclusively to the lightweight titanium-coated polypropylene mesh TiMesh that was used in 18 patients who underwent laparoscopic repair of large para-oesophageal hiatus hernia. TiMesh, weighing 35 g/m^2^ (poor size > 1.24 mm), was created by a German company, Medizintechnik GmbH, in Nuremberg [26]. The aim of the current study is to present the short-term outcomes for patients who have undergone laparoscopic hiatal hernia repair with a synthetic monofilament polypropylene mesh with adherent titanium dioxide surface coating (TiO2Mesh™) that was synthesized by a German company named BioCer Entwicklungs-GmbH [large pored mesh structure (2,8 mm) and 47 g/m^2^], which differs from the lightweight TiMesh by the presence of a biocompatible coating, blue orientation strips and the hydrophilic implant surface that supports the intraoperative handling and placement of TiO2Mesh™.

## Methods

### Patients

The study was performed retrospectively at Ramon y Cajal University Hospital, Spain, from December 2014 to October 2018. Data were collected on 27 consecutive patients with extensive hiatal hernia defects greater than 5 cm for which a laparoscopic repair was carried out by primary suture and extra reinforcement with a TiO_2_Mesh™.

### Preoperative workup

Each patient experienced a standard preoperative workup, including a physical examination, blood work, chest X-ray, barium swallow, oral endoscopy and computed tomography (CT) scan. In our institution, 24-h pH-metry and oesophageal manometry are not routinely indicated in LPHH.

### Surgical technique

**Fig. 1:**
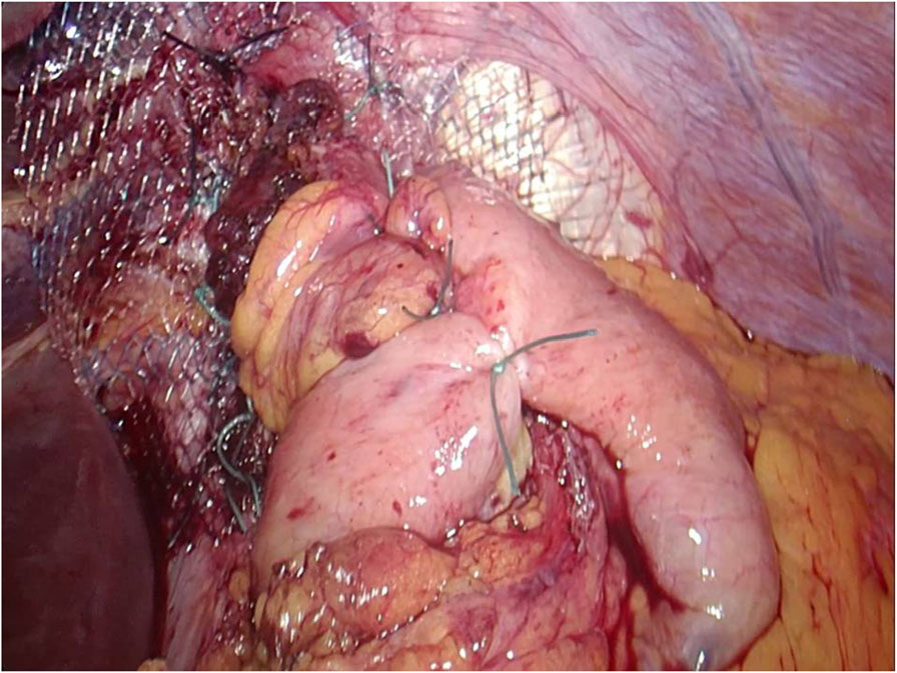
Nissen’s fundoplication after fixation of TiO2Mesh

Initially, the herniated sac with its contained stomach was reduced, the hernial sac was removed, and the phrenogastric ligament was dissected. Short gastric vessels were routinely transected. The gastrohepatic omentum was then separated, and the lower oesophagus was dissected from its surroundings. The crura were then repaired utilizing 3-4 interrupted nonabsorbable sutures (Ethibond 0) (Johnson & Johnson, Somerville, NJ, USA) between both diaphragmatic crura. Because of the large size of the hiatal defect (5 cm), a 7 cm × 10 cm manufactured monofilament polypropylene mesh with an adherent titanium dioxide surface covering (TiO2Mesh™) was placed and settled on the pillars of the oesophageal hiatus utilizing interrupted stitches on the edges of the mesh with non-absorbable sutures (Ethibond 2-0). A “floppy” Nissen or Toupet fundoplication was then customized in every patient utilizing nonabsorbable sutures (Ethibond 2-0) (Fig. 1).

### TiO_2_Mesh™ tissue reinforcement [25]

The TiO2Mesh™ tissue reinforcement is made of a synthetic monofilament polypropylene mesh thread and has a vast pored structure with blue orientation stripes. The mesh inserts are encompassed by a high-purity and adherent titanium dioxide surface covering to improve biocompatibility. Moreover, the lightweight character, together with both the large individual pores structure and the reduced material surface, promotes enhanced fibroblastic ingrowth and decreased shrinkage. Furthermore, its biocompatible covering is associated with a hydrophilic surface, and the blue orientation stripes are intended to encourage the intraoperative determination and fixation of TiO2Mesh™. This mesh is available in various standard sizes and shapes, and tailor-made mesh inserts for particular types of present-day hernia surgery are accessible. In our procedure, the mesh was custom-fitted specifically for large hiatal hernia (LHH) repair with a U-Shape of 7-cm × 10 cm.

### Postoperative course and long-term clinical assessment

Postoperatively, patients were put on a liquid diet (day 0 surgery) and discharged to go home on a soft diet for 10–15 days before returning to a typical diet. Follow-up was carried out at approximately 1 and 2 weeks and at the first, third, sixth and twelfth months; after that point, follow-up was conducted annually. A barium swallow and CT scan check were performed at the six- and twelve-month follow-ups. Oral endoscopy, 24-h pH-metry and oesophageal manometry were performed only if new symptoms occurred.

The investigation during the follow-up period was aimed at detecting radiological recurrence of hiatal hernia, mesh-related problems, gastroesophageal reflux (GERD) manifestations and dysphagia.

The best estimated value for radiological recurrence of hiatal hernia was the vertical extension of the stomach over the diaphragm by no less than 2 cm [23].

GERD symptom recurrence was defined by the assessment of acid reflux in postoperative 24-h pH-metry (DeMeester Scoring system > 14.7) and by the ingestion of proton pump inhibitor (PPI) medication to treat “de novo” symptoms of reflux.

### Statistical analysis

Statistical analysis was performed with SPSS 23.0 for Windows (IBM SPSS Inc., Chicago, IL, USA). Quantitative variables that were normally distributed were characterized by the mean and standard deviation. For non-Gaussian variable, the median and range were utilized. Categorical variables were characterized by the number and level of cases.

## Results

The study included 17 females (63%) and 10 males (37%), and the mean age was 73 years (range, 63–79 years). Assessment of patient risk was performed using the American Society of Anesthesiology (ASA) Scoring System [ASA I: 3 cases (11%), ASA II: 15 cases (56%), ASA III: 9 cases (33%)].

Nineteen cases were primary LPHH (70%), five cases were associated with gastric volvulus (18%), and 8 cases were large recurrent hiatal hernias (30%). The median surgical time was 120 min (range, 100–146 min) and overall hospital stay was 3 days (range, 2-3 days). A Nissen fundoplication was performed in 23 cases (85%), a Toupet in three cases (11%), and an angulation of the His angle in the last case (4%). One patient underwent conversion to open surgery (4%) due to bleeding of the lesser curvature after removal of the hernial sac. In one patient, laparoscopic incisional epigastric hernia repair was simultaneously performed. Intraoperative complications occurred in 5 patients (18%). Three patients developed a pneumothorax during dissection of the sac. However, they were managed intraoperatively without the placement of a chest tube. One patient had trocar site bleeding. Regarding postoperative complications (7%), there was 1 case of hyponatraemia, abdominal pain, anaemia and wall haematoma in an elderly patient with concomitant incisional hernia repair who was treated with a blood transfusion and pain killers, prolonging the hospital stay to 21 days. The patient who converted to open surgery developed a contained skin evisceration and was managed conservatively (primary reinforcement of skin with a running suture). In the remaining patients who underwent isolated LPHH repair, postoperative care was uneventful.

A complete follow-up assessment was obtained for all patients after a mean follow-up period of 18 months (range, 8-29 months). Regarding the most important items for assessment of the short-term outcome of TiO_2_Mesh™, 3 patients developed clinical recurrence of reflux symptoms (11%), assessed by the presence of acid reflux in 24-h pH-metry, and PPI treatment was added. There were two cases of radiological recurrence (7%). No mesh-related complications were found.

## Discussion

LHH repair remains a controversial and challenging intervention for most practising surgeons [1, 2]. Although repair with primary suture results in good clinical outcomes for the hiatal hernia defect, clinical and radiological recurrence have been reported in up to 33% of patients when treating these LPHH [3].

Evidence indicates that the utilization of mesh-strengthened hiatal repair has brought about a noteworthy decrease in recurrence rates in correlation with primary suturing of the hiatus, mainly in short-term follow-up [4–8]. Nevertheless, the standard of care for repairing LPHH remains controversial [1, 2, 9], particularly since no rules have been provided with respect to mesh position, type, method of fixation and indication [10].

To evaluate whether mesh type (biological or synthetic mesh) influences outcomes, Huddy et al. [27] published the results of a meta-analysis of four randomized controlled trials [22, 28–30] and five comparative studies [22, 23, 28, 29, 31] involving 676 patients undergoing LHH repair with a median follow-up ranging from 12-58 months using different techniques (primary suture vs. synthetic mesh vs. biological mesh). The authors showed that the recurrence rate was lower in patients receiving mesh implants (14.5% vs. 24.5%), with no statistically significant difference in complications between mesh and suture repair. However, when comparing synthetic mesh vs. suture repair, the recurrence rate was doubled in patients who did not receive mesh implants (12.6% vs. 24.6%), without any differences in complications (6.7% vs. 5.6%) or revisional surgery (5.9% vs. 6.7%). Interestingly, the rates of recurrence (17.1% vs. 23.4%), complications (12.5% vs. 15.6%) and reoperations (0% vs. 6.8%) were lower in the biological mesh group than in the suture repair group. However, and in spite of these good outcomes of biological meshes, not only costs are higher than the synthetic ones, but also, when compare overall recurrence rates, these were reduced in the synthetic mesh compared to biological mesh group (12.6% vs. 17.1%).

Simultaneously, reports from many other studies have encouraged the use of mesh-strengthened hiatal occlusion because of a considerable reduction in recurrence rates relative to those of suture repair alone [32–37] Despite these excellent outcomes, a few authors have reported increasing disappointment in the long-term outcomes of LPHH repair [23, 38].

The dangers of mesh-related complications, for example, migration of the mesh, oesophageal erosion, stenosis and postoperative dysphagia, have been the basis of contention against routine use of mesh reinforcement for several authors [11–13]^.^ We have recently reported our long-term results and complications identified with Crurasoft® mesh in 93 patients who underwent open or laparoscopic fundoplication for gastroesophageal reflux disease (GERD) or LPHH [V-moulded mesh with permeable PTFE on one side and extended polytetrafluorethylene (e-PTFE) on the other side (Bard® Davol INC)] repair for para-oesophageal hernias. In our investigation, the recorded mortality rate was 4% within the first 30 postoperative days. The reoperation rate was 5%, and the mesh was removed in 3 cases (3%). With a follow-up of 76 months, only 8 patients (9%) developed a repeated hiatal hernia [39]^.^ Of the five reoperation cases, one patient had oesophageal perforation that was probably secondary to the manipulation of the oesophagus rather than erosion of the mesh due to a prolonged surgical time. Two patients developed oesophageal and gastric perforation due to mesh erosion, and the last two patients were reoperated due to dysphagia related to fibrosis around the mesh. Mortality due to direct mesh-related complications was recorded in only 1 case due to oesophageal perforation if we excluded mortality that was secondary to pulmonary embolism, pneumonia, and multiple organ failure.

With a specific end goal to prevent or decrease this hazard, a few surgeons have stopped using synthetic mesh for another type of biologic and manufactured bio-absorbable meshes [14–22]^.^ However, the short- and long-term consequences of these studies were not as satisfactory as expected [23, 40–42]

In 2014, we published [20] our transient short-term results for laparoscopic repair of extensive para-oesophageal hiatal hernias with a manufactured mesh (Gore Bio A®). With a median follow-up of 20.3 months, only one out of 10 patients developed a recurrent hiatal hernia (10%), and no mesh-related complexities were observed. Unfortunately, we do not have long-term results; however, we concluded that the use of this mesh is safe and feasible, but the lack of long-term follow-up together with the small number of patients limits its use.

Currently, an ideal mesh for hiatal repair does not exist. Regardless of the considerable choice in available brands, almost all synthetic meshes for hernia surgery continue to use one of three essential materials: polyester, polypropylene and ePTFE. These materials are utilized alone or as part of a blend with extra elements. The ideal manufactured mesh ought to guarantee a high level of biocompatibility, be easy to handle and provide adequate fastness to avoid recurrence, shrinkage or degradation. To meet these criteria, more lightweight, large pored meshes have been produced recently, while assessing the dependability needed [24]^.^ In experimental investigations, an extra coating of nuclear titanium on the polypropylene fibres (TiO2Mesh™) has been shown to further increase the biocompatibility and reduce shrinkage rates compared with those of an indistinguishable polypropylene mesh without a titanium coating. Most of the studies mentioned the use of titanium in tacks for mesh fixation, although there is no available literature comparing titanium versus non-titanium mesh use in hernia repair. Therefore, our experience determined the biocompatibility of the titanium-coated polypropylene mesh [25]^.^

The TiO2Mesh™ tissue fortification is a manufactured monofilament polypropylene mesh thread and has a large pored structure with blue orientation stripes. The mesh inserts are encompassed by a high-purity and adherent titanium dioxide surface coating to improve biocompatibility. Together with its lightweight character, the large pored structure and the reduced material surface promote enhanced fibroblastic ingrowth and decreased shrinkage. Moreover, its biocompatible coating, together with a hydrophilic surface and blue orientation stripes, is designed to facilitate intraoperative handling and placement. Indeed, due to the high tensile strength of 55 N/cm, this mesh can be utilized for all regular open and laparoscopic systems of inguinal or incisional hernia repair, e.g., Lichtenstein strategy, transabdominal preperitoneal (TAPP) herniaplastic repair, total extraperitoneal (TEP) herniaplastic repair or all techniques for repairing ventral hernia [43–45].

To date, no trials or clinical investigations have been completed on this TiO2Mesh™ for the repair of hiatal hernias, and thus, no logical claims can be issued regarding this mesh. Therefore, the available literature relates only to the titanized polypropylene mesh TiMesh [26, 46–48]. Hazebroek et al. [26] described 18 patients who underwent LPHH repair with TiMesh, demonstrating postoperative complications in two patients (11%). Quality of life instrument (QOLRAD 5.79, *p* < 0.001) did not significantly change 2 years after hiatal hernia repair, and no differences were found between pre- and postoperative dysphagia scores. No indications of stricture development or prosthetic disintegration were observed by endoscopic evaluation at the follow-up time. One patient had a small (2 cm) sliding hiatal hernia detected by barium swallow, which was asymptomatic. Our results were comparable to the TiMesh in terms of recurrence rate and postoperative complication in the repair of large-sized hiatus hernias.

To maintain the properties of polypropylene [49] in terms of reducing the rate of recurrence and avoiding the mesh-related complications of these kinds of meshes, we decided to change our preferred mesh from Crurasoft to TiO_2_Mesh™. Our preliminary results appear to be highly satisfactory. Intraoperatively, we consider the TiO_2_Mesh™ to be excellent regarding handling, placement and consistency in comparison to Crurasoft and Gore BioA mesh. In the present study, the postoperative course related to LPHH repair was uneventful, with a median of 3 days of hospital stay without any vomiting or postoperative dysphagia. Moreover, with a follow-up of 18 months, the radiological recurrence rate was 7%. Unfortunately, in 3 patients (11%), “de novo” GERD symptoms were detected in 24-h pH-metry. All patients were on PPIs, but one of them had to be re-operated due to uncontrollable GERD symptoms without PPIs 14 months after primer surgery. In this case, wrap migration through the hiatus was ruled out during the intervention. Mesh was correctly integrated in the hiatus, and symptoms were related more to a disruption of the Nissen fundoplication. For this reason, a new Nissen fundoplication was performed, and the patient is currently asymptomatic 4 months after this second surgery. The remaining patients were controlled with PPIs.

Because this retrospective study had a small number of cases, a short duration of follow-up, no direct comparison mesh evaluated other than by historical controls, there were some limitations, but our preliminary results with this type of mesh are encouraging. Moreover, this study is the first to be published describing results with TiO_2_Mesh™ regarding the repair of LPHH.

## Conclusion

The use of TiO_2_Mesh™ for the laparoscopic repair of large para-oesophageal and recurrent hiatal hernias was safe and had an acceptably low recurrence rate of GERD in the short evaluation period. Long-term studies will be important for evaluating whether this new manufactured mesh is not only safe but also effective in preventing recurrence.

## Data Availability

The datasets used and/or analysed during the current study are available from the corresponding author on reasonable request.

## Abbreviations

CT: Computerized Tomography
ePTFE: expanded Polytetrafluoroethylene
GERD: Gastroesophageal Reflux Disease
LPHH: Large Paraesophageal Hiatal Hernias
PH: Potential hydrogen
PTFE: Polytetrafluoroethylene
PVDF: Polyvinylidene Fluoride
QOLRAD: Quality-of-Life in Reflux and Dyspepsia
TAPP: Transabdominal Preperitoneal Hernioplasty
TEP: Total Extraperitoneal Hernioplasty
